# WEAK CROSSABILITY BARRIER BUT STRONG JUVENILE SELECTION SUPPORTS ECOLOGICAL SPECIATION OF THE HYBRID PINE *PINUS DENSATA* ON THE TIBETAN PLATEAU

**DOI:** 10.1111/evo.12496

**Published:** 2014-09-22

**Authors:** Wei Zhao, Jingxiang Meng, Baosheng Wang, Lisha Zhang, Yulan Xu, Qing-Yin Zeng, Yue Li, Jian-Feng Mao, Xiao-Ru Wang

**Affiliations:** 1 State Key Laboratory of Systematic and Evolutionary Botany, Institute of Botany, Chinese Academy of SciencesBeijing, 100093, China; 2 University of Chinese Academy of SciencesBeijing, 100049, China; 3 National Engineering Laboratory for Forest Tree Breeding, Key Laboratory for Genetics and Breeding of Forest Trees and Ornamental Plants of Ministry of Education, Beijing Forestry UniversityBeijing, 100083, China; 4 Department of Ecology and Environmental Science, Umeå UniversitySE-901 87, Umeå, Sweden; 5 Hebei Academy of Forestry ScienceShijiazhuang, 050064, China; 6 Key Laboratory for Forest Resources Conservation and Use in the Southwest Mountains of China, College of Forestry, Southwest Forestry UniversityKunming, 650224, China

**Keywords:** Cross-compatibility, ecological selection, hybrid speciation, local adaptation, population divergence, transplantation experiment

## Abstract

Determining how a new hybrid lineage can achieve reproductive isolation is a key to understanding the process and mechanisms of homoploid hybrid speciation. Here, we evaluated the degree and nature of reproductive isolation between the ecologically successful hybrid species *Pinus densata* and its parental species *P. tabuliformis* and *P. yunnanensis*. We performed interspecific crosses among the three species to assess their crossability. We then conducted reciprocal transplantation experiments to evaluate their fitness differentiation, and to examine how natural populations representing different directions of introgression differ in adaptation. The crossing experiments revealed weak genetic barriers among the species. The transplantation trials showed manifest evidence of local adaptation as the three species all performed best in their native habitats. *Pinus densata* populations from the western edge of its distribution have evolved a strong local adaptation to the specific habitat in that range; populations representing different directions of introgressants with the two parental species all showed fitness disadvantages in this *P. densata* habitat. These observations illustrate that premating isolation through selection against immigrants from other habitat types or postzygotic isolation through selection against backcrosses between the three species is strong. Thus, ecological selection in combination with endogenous components and geographic isolation has likely played a significant role in the speciation of *P. densata*.

Hybridization is recognized as an important force in plant evolution (Anderson and Stebbins 1954; Grant 1981; Arnold 1997; Rieseberg 1997). One outcome of hybridization is the generation of new species either through allopolyploid or homoploid hybrid speciation (HHS) (Abbott 1992). Allopolyploid hybrid speciation is a rapid process as the hybrid achieves instantaneous reproductive isolation from the parental species due to differences in ploidy, which results in a strong compatibility barrier. In contrast, homoploid hybrids lack an effective isolation mechanism to overcome gene flow from their parental species. In the absence of an isolation barrier, the new hybrid lineages are likely vulnerable to extinction by competition and/or gene flow from their parental species (Buerkle et al. 2000; Wolf et al. 2001). Thus, the development of reproductive isolation between a newly stabilized hybrid lineage and its parental species is the most difficult and important step of HHS.

Reproductive barriers are usually classified as pre- or postzygotic. Premating prezygotic barriers typically result from geographical, phenological, ecological, and/or behavioral differences that reduce interspecific matings. Postmating prezygotic barriers include interactions between the male and female gametes during the formation of hybrid zygotes (e.g., pollen–stigma recognition), and the postmating postzygotic barriers include intrinsic genetic factors that reduce hybrid viability (Coyne and Orr 2004; Rieseberg and Willis 2007). Ecology can also act as an extrinsic postzygotic reproductive barrier by reducing the mating success and fitness of hybrids and their descendants (Schluter 2001; Rieseberg et al. 2003). Characterizing the various components that contribute to the reproductive isolation between hybrid lineages and their parental species is important for understanding the process of HHS.

*Pinus densata* represents a highly ecologically successful case of HHS in plants. The species forms extensive forests on the southeastern Tibetan Plateau. DNA marker based analyses suggest that *P. densata* originated from hybridization between *P. tabuliformis* and *P. yunnanensis* in the late Miocene (Wang and Szmidt 1994; Wang et al. 2011; Gao et al. 2012). *Pinus tabuliformis* is widely distributed across northern and central China, and *P. yunnanensis* has a relatively limited range in southwestern China. The distribution of the three pine species forms a geographical succession, with *P. tabuliformis*, *P. densata*, and *P. yunnanensis* generally found in northerly, intermediate, and southerly latitudes, respectively (Mao and Wang 2011). Previous studies hypothesized that the evolution of *P. densata* following the initial hybridization events into a stabilized taxonomic unit was promoted by uplift of the Tibetan Plateau, after which the successful hybrid lineages colonized the new, empty plateau habitat that was inaccessible to both parental species (Wang and Szmidt 1994; Wang et al. 2001; Ma et al. 2006). An ancestral hybrid zone between the two parental species has been identified in the northeastern periphery of *P. densata*'s range, from which the species colonized the plateau by stepwise westward migration (Wang et al. 2011; Gao et al. 2012). The distribution of *P. densata* can be divided into three regions: eastern, central, and western. The eastern populations are adjacent to *P. tabuliformis* populations and have experienced strong pollen-mediated introgression from them, whereas the central populations have received gene flow from *P. yunnanensis* (Wang et al. 2011). In contrast, western populations of *P. densata* have been isolated from both parental species for ca. 0.3 million years (Gao et al. 2012), resulting in the evolution of unique mitochondrial and chloroplast (cp) haplotype compositions (Wang et al. 2011; Gao et al. 2012). Thus, we can regard the populations in these three regions as three distinct genetic groups: the eastern and central groups represent introgressants (backcrosses) with *P. tabuliformis* and *P. yunnanensis*, respectively, whereas the western group represents a stabilized advanced generation of *P. densata* with little influence from parental species. Ecological niche modeling was indicative of distinct niche divergence in *P. densata*, and showed that local adaptation and geographic barriers may have contributed to the differentiation and reproductive isolation in the species complex (Mao and Wang 2011). However, the degree and nature of reproductive isolation between *P. densata* and its parental species remain to be empirically identified and defined.

In this study, we first performed controlled crosses among the three species to assess their crossability as a measurement of postmating zygotic barriers. Second, we conducted reciprocal transplantation experiments to evaluate the fitness differentiation among these species. Third, we examined the genetic relationships of the populations included in the crossing and transplantation experiments to explore whether and how natural populations representing different directions of introgression differ in terms of adaptation in different habitats. Divergence in adaptation can act as a premating barrier by preventing immigrants from establishing in non-native environments. It can also strengthen postzygotic isolation by eliminating unfit backcrosses. Our objectives were to evaluate the postmating genetic barriers among the three pine species, and explore the degree and patterns of adaptive divergence at the early life stages (seedling) in the species complex. Such information is essential for understanding the intrinsic and extrinsic factors that contribute to the reproductive isolation of the *P. densata* complex and the mechanisms of hybrid speciation and species divergence across ecological transitions.

## Materials and Methods

### INTERSPECIFIC CROSSES

**Figure 1:**
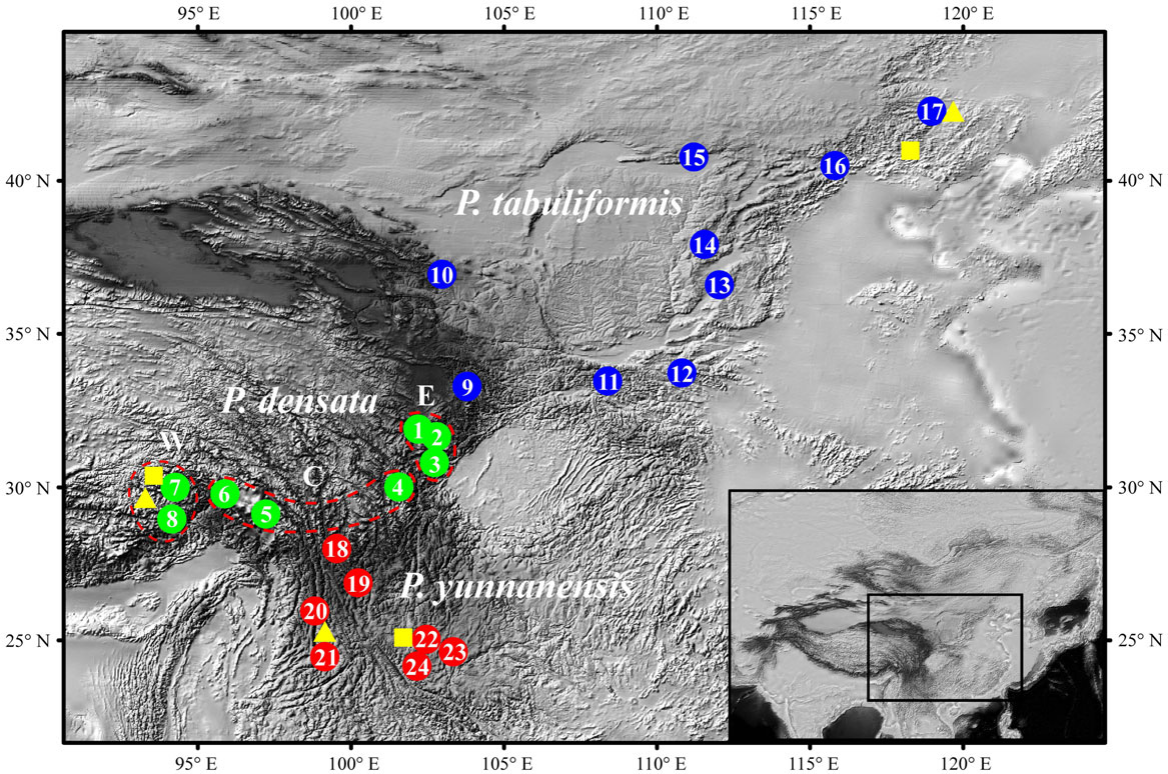
Geographic distribution of the 24 populations included in the transplantation experiments. The three *P. densata* groups, east, central, and west, identified by previous DNA marker analyses are designated E (population nos. 1–3), C (population nos. 4–6), and W (population nos. 7 and 8), respectively. *Pinus tabuliformis* populations are nos. 9–17, and *P. yunnanensis* populations are nos. 18–24. Sites for crossing and transplantation experiments are indicated by triangles and squares, respectively.

To test the crossability among the three pine species, we performed controlled reciprocal crosses between species in the wild from 2006 to 2011. Crosses were accumulated over four pollination seasons to achieve a reasonable level of balance in the various mating combinations. One representative stand of each species (Heilihe, 41°34′N, 118°23′E for *P. tabuliformis*; Baoshan, 25°08′N, 99°08′E for *P. yunnanensis*; and Linzhi, 29°44′N, 94°18′E for *P. densata*) was selected for the crossing experiment (Table1; Fig.1). In each stand, 15–30 trees were selected as maternal parents and 4–5 trees as pollen parents. No selection criteria were applied in choosing parent trees other than that they were producing male and female strobili. On each maternal tree, 20–40 female strobili were isolated with paper bags before receptivity. Hand pollination was applied at the maximum receptivity of the female strobili. Open pollinated cones on each maternal tree were used as controls. The reason for using open pollination (OP) rather than intraspecific crosses as control was dictated by the number of female strobili available (and reachable) on each maternal tree, which limits the possibility of conducting both intra- and interspecific crosses on the same tree. The advantage of OP is that it gives a better representative estimation of intraspecific mating compatibility, but the disadvantage is that it represents a different pollination environment than the controlled crosses (see also Results and Discussion). In addition, we also selected one tree in each stand during each pollination season on which female strobili were bagged but not pollinated. The isolation bags were removed three weeks after pollination. All fully developed crossed cones and five open pollinated cones were collected from each maternal tree when they were mature.

**Table 1:** Interspecific crosses performed in this study

	Experiment	Maternal	Paternal	Pollen	No. of	No. of	No. of	No. of harvested	No. of analyzed
Year	site	species	species	source	crosses	isolation bags	pollinated strobili	hybrid cones	cones (cross/OP)
2006	Heilihe	*P. tabuliformis*	*P. densata*	Linzhi	23	666	>1000	397	397/52
2008	Heilihe	*P. tabuliformis*	*P. yunnanensis*	Kunming	27	651	>1000	356	356/48
2010	Heilihe	*P. tabuliformis*	*P. yunnanensis*	Baoshan	15	779	>1100	175	58/65
2010	Baoshan	*P. yunnanensis*	*P. densata*	Linzhi	14	330	>700	597	64/30
2010	Baoshan	*P. yunnanensis*	*P. tabuliformis*	Heilihe	22	355	>700	408	100/79
2010	Linzhi	*P. densata*	*P. yunnanensis*	Baoshan	14	390	>700	115	53/36
2011	Linzhi	*P. densata*	*P. tabuliformis*	Heilihe	18	463	>1000	540	66/67
				Total	133	3634	>6000	2588	1094/377

OP = open pollination.

In pines, the seed cones take 1.5–3 years (depending on species) to mature after pollination (Mirov 1967). The length of this process is due to the fact that after pollination, the pollen tube grows slowly through the nucellar tissue and enters a state of dormancy during winter; fertilization takes place in the second year (McWilliam 1959; Fernando et al. 2005). After fertilization, the seed cones take ≥5 months to develop and reach maturity. The pollination season in the *P. tabuliformis* site Heilihe ranged from mid-May to early June, and cones matured in October of the following year. In the *P. densata* site Linzhi, the pollination season ranged from early to mid-May and cones matured in October to November of the next year. In *P. yunnanensis* site Baoshan, the pollination season was from early to mid-March and cones matured 22 months later. Due to the lack of overlapping flowering phenology among the three species (experimental stands), and for operational feasibility, pollen used in crossing experiments were collected and extracted during the previous season, dried and stored at −20°C in sealed bottles. Prior to pollination, the viability of the pollen was assessed in vitro, and only pollen with a high germination rate (≥85%) was used in the pollination experiments.

### CONE ANALYSIS

The crossed and open-pollinated cones were analyzed following the system described in Mao et al. (2009). Briefly, the scales in a seed cone were divided into fertile and infertile scales; each fertile scale contained two ovules at its base. Twice the number of fertile scales in a cone determines its maximum potential seed production. During cone development, some ovules are aborted and some develop into full-sized seeds, which consist of both filled and empty seeds. The proportion of full-sized seeds in a cone is defined as the number of full-sized seeds relative to seed potential (SP) of the cone. Seed efficiency is defined as the number of filled seeds relative to SP.

For each cone, we measured two seed and three cone morphometric traits and five seed production traits. The morphometric traits included seed length, seed weight, cone length, total number of scales per cone, and number of fertile scales per cone. The seed production traits included number of full-sized seeds per cone, number of filled seeds per cone, ratio of filled seeds in the full-sized seeds, proportion of full-sized seeds, and seed efficiency. The differences among interspecific crosses and OP in the morphometric and seed production traits were evaluated using the nonparametric Kruskal–Wallis multiple-range test in agricolae (De Mendiburu 2009). To partition the variance in these 10 traits between maternal and paternal effects at species and genotype levels (not including OP), and to evaluate the overall statistical significance of the differences, we performed a permutational ANOVA implemented in vegan (Oksanen et al. 2013). In this analysis, genotype was nested within species. All statistical tests were performed in *R*.

### QUANTIFICATION OF CROSSABILITY

Crossability in this study refers to the ease with which two species can be successfully crossed compared to OP of the maternal parents. In the majority of *Pinus* species, unpollinated ovules abort soon after the pollination season, and the growth of pollen tubes in the nucellus is necessary for the continued development of ovules (McWilliam 1959). The filled seeds develop from the fertilized ovules without embryo mortality, thus reflecting mating success. The degree of crossability between pine species has been defined traditionally (Critchfield 1967) as:
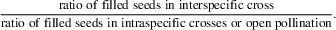


This frame of comparison considers only fully developed seeds. In pines (especially in the subgenus *Pinus*), hybrid failure can occur due to the inability of pollen tubes to grow and function normally in the nucellar tissue of the foreign species, which causes the breakdown of developing megagametophytes and ovule collapse (McWilliam 1959). Thus, a large portion of the ovules can abort due to incompatibility. These aborted ovules were not accounted for in previous crossability measurements. In this study, we defined the crossability between two species as:
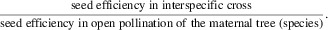


Because our seed efficiency was defined as the number of filled seeds relative to the SP (i.e., 2× no. of fertile scales), the loss due to ovule abortion is considered. Assessment of crossability based on SP is more appropriate than that based on only fully developed seeds, but requires much tedious cone analysis to count the fertile scales in each cone.

### TRANSPLANTATION EXPERIMENTS

To estimate the relative fitness of the three species outside their respective native habitat, we established three transplantation trials in three locations; each represents a native habitat for each of the three species. Site Pingquan (40°59′N, 118°26′E) is in the north central range of *P. tabuliformis* distribution, site Linzhi (29°40′N, 94°20′E), Tibet, represents the native habitat for *P. densata*, and site Kunming (25°04′N, 102°46′E) is in the central distribution of *P. yunnanensis* (Fig.1). We collected bulked seeds from 24 populations to represent the distribution of the three species. For *P. tabuliformis*, nine populations (nos. 9–17, Fig.1) were included, which spanned a range from 33°N to 42°N (Table S1); for *P. yunnanensis*, seven populations (nos. 18–24) were included with origins from 24°N to 28°N (Fig.1, Table S1). The selection of *P. densata* populations was guided by our previous DNA marker based analyses (Wang et al. 2011; Gao et al. 2012), which showed that the distribution of *P. densata* could be divided into eastern, central, and western regions. We included three (nos. 1–3), three (nos. 4–6), and two populations (nos. 7–8) from each of the regions, respectively, in the transplantation experiments (Fig.1).

The three trials were established in spring 2011 using random block designs, with 60 seeds (50 in Linzhi) in each population plot and four blocks (five in Kunming) for each experimental site. The trials were monitored and watered every second day during the seed germination stage. After that, no other cultivation activities were applied to the seedlings, with the exception of weeding, until spring 2013.

A general aspect of local adaptation in conifer trees is their ability to maximize growth and avoid frost damage by synchronizing growth phenology (e.g., terminal bud set) with the local climate (Howe et al. 2003). Thus, we measured seed germination rates during the first spring, and survival and seedling height of each population in the late spring and autumn of each year until the third spring. We registered the proportion of seedlings of each population that formed the terminal bud by the first week of October in Linzhi and by the first week of November in the Pingquan and Kunming sites. Bud set was scored only once for the first year seedlings in the fall of 2011. Survival is a direct measure of fitness, and growth is the most important fitness component at the seedling stage. We used the product of survival rate and seedling height of each population as an approximation of population biomass and thus a coarse measure of population performance (fitness) at each site. The relative performance (fitness) of each population at a site was assessed by comparing this product value to that of the local population (i.e., the value of the local population is set to 1). The mean and standard error in germination rate, survival, height, bud set, and the relative fitness were calculated for each population at each testing site. Least significant difference multiple-range tests were used to detect significant mean differences between species (groups for *P. densata*) in these traits using agricolae (De Mendiburu 2009).

### POPULATION GENETIC DIFFERENTIATION

We estimated the degree of divergence between the three groups of *P. densata* and the two parental species using cp and nuclear DNA polymorphism data. The cpDNA data were extracted from Wang et al. (2011), which consisted of genotypes over five microsatellite (cpSSR) loci for 496 individuals from 20 populations used in the present crossing and common garden experiments (Table S1). Nuclear DNA data were extracted from Gao et al. (2012), which contained sequences of eight gene loci for 193 individuals from 16 populations included in the current experiments (Table S1). Based on the cpDNA and nuclear DNA data, we calculated the pairwise population differentiation (*F_ST_*) between groups of populations using Arlequin version 3.1 (Excoffier et al. 2005), with significance testing based on 10,000 permutations.

## Results

### INTRASPECIFIC OP VERSUS INTERSPECIFIC CROSSES

A total of 133 combinations of interspecific crosses were performed (64 between *P. tabuliformis* and *P. yunnanensis*, 41 between *P. densata* and *P. tabuliformis*, and 28 between *P. densata* and *P. yunnanensis*), yielding more than 2500 hybrid cones, of which 1094 cones were measured and analyzed individually for the five morphometric and five seed production traits (Table1). From the mother trees used in the interspecific crosses, 377 open pollinated cones were also analyzed. The unpollinated controls produced no mature cones because all conelets aborted in the first year. Parthenocarpy was not observed in any of the three species.

**Figure 2:**
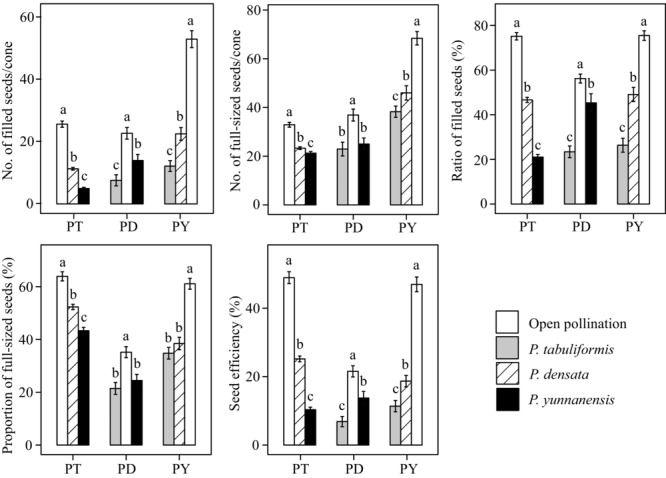
Error-bar chart (mean ± SE) for the five seed production traits, number of filled seeds per cone, number of full-sized seeds per cone, ratio of filled seeds in the full-sized seeds, proportion of full-sized seeds, and seed efficiency in interspecific crosses and OP. Maternal species *Pinus tabuliformis, P. densata*, and *P. yunnanensis* are denoted by PT, PD, and PY, respectively, on the x-axis. Paternal species or treatment are indicated by open (open pollination), gray (*P. tabuliformis*), diagonal (*P. densata*), and black (*P. yunnanensis*) bars. Different letters (a, b, and c) above each bar indicate significant differences (*P* = 0.05) among open pollination and interspecific crosses based on Kruskal–Wallis multiple-range tests.

Differences in the 10 traits between interspecific crosses and OP of each maternal species were summarized in Figures2 and S1. For the five seed and cone morphometric traits, the difference among maternal species was large, whereas the paternal effect on these traits was relatively small (although significant in a few cases, Fig. S1). In general, we concluded that OP and interspecific crosses did not differ much in these morphometric traits for any maternal species. By contrast, for the five seed production traits, significant differences were observed between OP and interspecific crosses in all three maternal species (Fig.2). In general, OP performed significantly better than the interspecific crosses; for example, the average seed efficiency in OP of *P. tabuliformis*, *P. densata*, and *P. yunnanensis* was 48.91%, 21.54%, and 46.96%, respectively, as compared to 10.33–25.16%, 6.85–13.74%, and 11.37–18.68% in interspecific crosses of each species (Fig.2). Among the interspecific combinations, crosses between *P. tabuliformis* and *P. yunnanensis* showed the largest declines in seed efficiency compared to their OP (i.e., 38.58% decrease for *P. tabuliformis* × *P. yunnanensis* vs. OP in *P. tabuliformis*, and 35.59% decrease for *P. yunnanensis* × *P. tabuliformis* vs. OP in *P. yunnanensis*).

### INTERSPECIFIC CROSSES AND CROSSABILITY

Because cones from both OP and controlled cross-pollination of the same maternal parent showed comparable potential for seed set (i.e., no. of fertile scales, cone length, Fig. S1), the observed difference in seed production traits between the two pollination categories was therefore ascribable to mating combinations rather than to inferior cone development due to bagging. This presumption is in general agreement with what has been reported for conifers in which the differences in seed production between OP and intraspecific controlled crosses are not significant (Park and Fowler 1984; Kormutak et al. 1992). To explore the maternal and paternal effects on the interspecific crosses, we partitioned the variance in each trait to between and within species between genotype components. For the five seed and cone morphometric traits, the majority of variance was distributed among maternal species (59.46%, 69.53%, 31.07%, 69.33%, and 77.59%, respectively), and the paternal influence on these traits was small, although significant on some of them (Table2).

**Table 2:** ANOVA for the 10 morphometric and seed production traits in interspecific crosses among *Pinus densata*, *P. tabuliformis*, and *P. yunnanensis*

Traits	Source	df	SS	*F*	Percentage of variance
Seed length (cm)	Maternal species	2	9.12	1190.10***	59.46
	Paternal species	2	0.32	41.09***	2.05
	Paternal genotype	25	0.56	5.84***	3.65
	Maternal genotype	68	1.54	5.90***	10.01
	Residuals	994	3.81		24.83
Weight of 100 seeds (g)	Maternal species	2	1720.00	1297.80***	69.53
	Paternal species	2	18.70	14.10***	0.76
	Paternal genotype	25	71.90	4.34***	2.91
	Maternal genotype	62	119.00	2.90***	4.81
	Residuals	821	544.00		21.99
Cone length (cm)	Maternal species	2	305.00	458.03***	31.07
	Paternal species	2	0.75	1.12	0.08
	Paternal genotype	25	94.60	11.38***	9.65
	Maternal genotype	68	248.00	10.99***	25.35
	Residuals	998	332.00		33.85
No. of total scales/cone	Maternal species	2	480,000.00	1997.60***	69.33
	Paternal species	2	460.00	1.91	0.07
	Paternal genotype	25	29,200.00	9.73***	4.22
	Maternal genotype	68	62,800.00	7.69***	9.07
	Residuals	998	120,000.00		17.32
No. of fertile scales/cone	Maternal species	2	212,000.00	3202.5***	77.59
	Paternal species	2	1620.00	24.6***	0.6
	Paternal genotype	25	6750.00	8.2***	2.47
	Maternal genotype	68	19,800.00	8.8***	7.24
	Residuals	998	33,000.00		12.09
No. of full-sized seeds/cone	Maternal species	2	50,100.00	198.266***	15.45
	Paternal species	2	3150.00	12.461***	0.97
	Paternal genotype	25	38,200.00	12.114***	11.8
	Maternal genotype	68	107,000.00	12.413***	32.89
	Residuals	998	126,000.00		38.89
No. of filled seeds/cone	Maternal species	2	9310.00	77.021***	6.29
	Paternal species	2	13,500.00	111.546***	9.11
	Paternal genotype	25	18,300.00	12.088***	12.33
	Maternal genotype	68	46,700.00	11.363***	31.54
	Residuals	998	60,300.00		40.73
Ratio of filled seeds (%)	Maternal species	2	473.00	0.537	0.06
	Paternal species	2	158,000.00	179.854***	18.93
	Paternal genotype	25	87,200.00	7.939***	10.45
	Maternal genotype	68	151,000.00	5.039***	18.03
	Residuals	998	439,000.00		52.53
Proportion of full-sized seeds/cone (%)	Maternal species	2	74,200.00	115.032***	11.63
	Paternal species	2	16,100.00	24.995***	2.53
	Paternal genotype	25	101,000.00	12.500***	15.8
	Maternal genotype	68	125,000.00	5.698***	19.59
	Residuals	998	322,000.00		50.45
Seed efficiency (%)	Maternal species	2	6870.00	18.727***	2.13
	Paternal species	2	44,900.00	122.554***	13.93
	Paternal genotype	25	37,700.00	8.235***	11.7
	Maternal genotype	68	50,000.00	4.010***	15.5
	Residuals	998	183,000.00		56.73

*** *P* < 0.001.

On the five seed production traits, a stronger paternal influence was evident and the maternal and paternal effects varied among the traits. For the number of full-sized seeds per cone, number of filled seeds per cone, and proportion of full-sized seed, the joint maternal effect (i.e., maternal species and maternal genotype, 31.22–48.34%) was larger than the joint paternal effect (12.77–21.44%). For the ratio of filled seeds and seed efficiency, the paternal component was larger, as paternal species and genotypes jointly accounted for 29.38% and 25.63% of the total variance, whereas the joint maternal effect was 18.09% and 17.63%, respectively. On these five seed production traits, the residual component was large, accounting for 38.89–56.73% (Table2).

**Figure 3:**
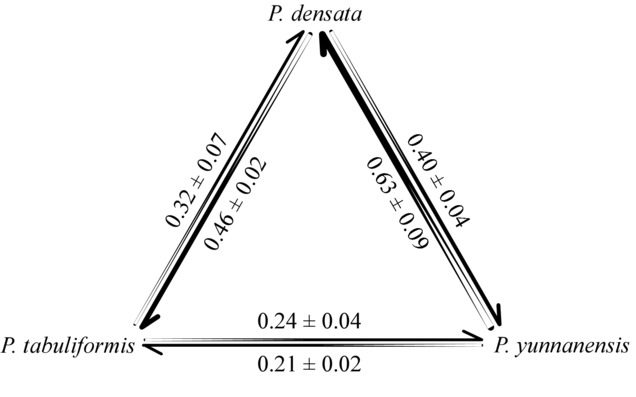
Interspecific crossabilities (mean ± SE) among *Pinus tabuliformis, P. densata*, and *P. yunnanensis*. Arrows start from paternal species and toward maternal species.

The degree of crossability varied considerably among species combinations, and between the directions of reciprocal crosses in two species-pair combinations (Fig.3). Patterns of crossability among species were similar using the two estimation methods (Table S2). Thus, only results estimated from seed efficiency are presented. The mean crossability between the *P. tabuliformis* and *P. yunnanensis* was 0.23, between *P. densata* and *P. tabuliformis* was 0.39, and between *P. densata* and *P. yunnanensis* was 0.51. Hence, the postmating crossing isolation was strongest between *P. tabuliformis* and *P. yunnanensis*, intermediate between *P. densata* and *P. tabuliformis*, and weakest between *P. densata* and *P. yunnanensis*. Significant differences were found between the reciprocal crosses of *P. densata* and *P. tabuliformis* (0.32 for *P. densata* × *P. tabuliformis* vs. 0.46 for *P. tabuliformis* × *P. densata*; *P* < 0.01), and of *P. densata* and *P. yunnanensis* (0.63 for *P. densata* × *P. yunnanensis* vs. 0.40 for *P. yunnanensis* × *P. densata*; *P* < 0.01).

### TRANSPLANTATION EXPERIMENTS

We measured four traits in 24 populations across three common garden sites; the seed germination rate, survival rate, seedling height, and bud set. At the *P. tabuliformis* site Pingquan, seed germination rates of *P. tabuliformis* populations (72.64 ± 2.06%) were much higher than that of *P. yunnanensis* (47.74 ± 4.18%) and *P. densata* central and western populations (40.69 ± 2.89% and 41.67 ± 2.08%, respectively; Table3). *Pinus densata* eastern populations (nos. 1–3), on the other hand, had a similar germination and growth rate as *P. tabuliformis*, although their survival decreased more than did that of *P. tabuliformis* (Table S3). Among the three *P. densata* eastern populations, one population Baoxing (no. 3) deviated from the other two in performance, as it was almost eliminated by the third year (survival rate 2.50%), whereas the other two had 42.92% and 47.92% survival (Fig. S2; Table S4). This population is unique in terms of its cytoplasmic DNA compositions, which is suggestive of geographical isolation from adjacent populations (Wang et al. 2011). Whether such isolation to a specific site and/or its unique cytoplasmic DNA composition has contributed to its divergent adaptation remains unclear. The other two groups of *P. densata* and all populations of *P. yunnanensis* were dead after the first winter (Table S3). Large variations in survival and growth among populations of *P. tabuliformis* were also observed at this site. Such variations are expected considering the wide geographical range of the collected populations, from 33°N to 42°N. In general, southern populations of *P. tabuliformis* (e.g., no. 9, 33°N and no. 11, 33°N) grew more poorly than the species average at this site (Fig. S2; Table S4).

**Table 3:** Germination rate, ratio of bud set and the third year survival, seedling height and relative fitness of each species or group of populations at the *Pinus tabuliformis* home site Pingquan, *P. densata* home site Linzhi, and *P. yunnanensis* home site Kunming

Site	Species	Germination rate	Bud set ratio	Third-year survival (%)	Third-year height (cm)	Third-year relative fitness
Pingquan	*P. tabuliformis*	72.64 ± 2.06^a^	92.44 ± 0.87^a^	44.03 ± 2.96^a^	21.16 ± 1.65^a^	0.90 ± 0.11^a^
	*P. densata* east	73.89 ± 3.87^a^	82.48 ± 5.97^ab^	31.11 ± 14.38^a^	18.40 ± 4.23^a^	0.64 ± 0.31^a^
	*P. densata* central	40.69 ± 2.89^b^	73.03 ± 1.99^b^	0 ± 0^b^	na	0 ± 0^b^
	*P. densata* west	41.67 ± 2.08^b^	51.26 ± 0.53^c^	0 ± 0^b^	na	0 ± 0^b^
	*P. yunnanensis*	47.74 ± 4.18^b^	11.40 ± 7.73^d^	0 ± 0^b^	na	0 ± 0^b^
Linzhi	*P. tabuliformis*	82.06 ± 2.66^a^	85.87 ± 7.39^a^	9.56 ± 1.04^b^	12.88 ± 1.23^ab^	0.28 ± 0.05^b^
	*P. densata* east	74.50 ± 7.51^ab^	66.38 ± 33.19^a^	12.17 ± 4.92^ab^	11.04 ± 1.08^b^	0.31 ± 0.15^b^
	*P. densata* central	60.83 ± 6.41^b^	10.59 ± 4.56^b^	6.83 ± 3.38^b^	12.51 ± 1.09^ab^	0.20 ± 0.11^b^
	*P. densata* west	71.50 ± 0.5^ab^	0.38 ± 0.38^b^	19.75 ± 4.25^a^	17.41 ± 1.75^a^	0.76 ± 0.24^a^
	*P. yunnanensis*	62.86 ± 4.70^b^	3.57 ± 3.57^b^	7.57 ± 1.94^b^	12.89 ± 1.54^ab^	0.22 ± 0.07^b^
Kunming	*P. tabuliformis*	89.07 ± 2.00^a^	93.11 ± 1.33^a^	83.84 ± 1.98^a^	15.71 ± 0.93^b^	0.60 ± 0.03^b^
	*P. densata* east	83.56 ± 6.46^a^	69.73 ± 23.49^a^	76.25 ± 7.34^a^	20.01 ± 0.63^ab^	0.70 ± 0.07^ab^
	*P. densata* central	76.11 ± 10.06^a^	17.60 ± 8.92^b^	70.83 ± 10.48^a^	20.71 ± 3.47^ab^	0.70 ± 0.18^ab^
	*P. densata* west	61.83 ± 8.50^b^	7.12 ± 0.53^b^	54.17 ± 7.50^b^	21.13 ± 0.98^ab^	0.53 ± 0.10^b^
	*P. yunnanensis*	88.90 ± 1.00^a^	2.71 ± 2.59^b^	78.23 ± 1.23^a^	27.80 ± 3.28^a^	1.00 ± 0.12^a^

Means with different superscript letters (a, b, c, and d) are significantly different (*P* = 0.05) in least significant difference multiple-range tests. na = data not available due to mortality.

At the *P. densata* site Linzhi, the seedlings of all three species suffered heavy mortality during the first year, with average mortality rates of 70.95%, 60.17%, 53.16%, 43.75%, and 53.29% for *P. tabuliformis*, *P. densata* east, *P. densata* central, *P. densata* west, and *P. yunnanensis*, respectively (Tables3 and S3). The germination rate of *P. densata* west (71.50%) was much higher in this native habitat than in Pingquan and Kunming (41.67% and 61.83%, respectively; Table3). It also showed higher survival and growth than the other species and groups over the seasons. This resulted in a considerable advantage (2.5- to 3.8-fold) by the third year in gross population performance at this site for the *P. densata* local group over all other species and groups (Table3).

At the *P. yunnanensis* site Kunming, all *P. tabuliformis, P. yunnanensis* and *P. densata* eastern and central populations showed high germination rates (89.07% 88.90%, 83.56%, and 76.11%, respectively), and only *P. densata* western populations showed relatively poor germination (61.83%; Table3). At this site, all populations had stable survival over the seasons (Tables S3 and S4). By the third spring, survival rates were 83.84 ± 1.98%, 78.23 ± 1.23%, 76.25 ± 7.34%, 70.83 ± 10.48%, 54.17 ± 7.50% for *P. tabuliformis*, *P. yunnanensis*, *P. densata* east, central, and west, respectively. In growth, *P. yunnanensis* populations had an advantage over the others; by the third year, the height of *P. yunnanensis* was 131.57–176.96% compared to the other species (Table3).

The bud set ratio of the populations in the three testing trials showed clear latitudinal clines (Table3). In the northern site Pingquan, 92.44% of *P. tabuliformis* seedlings formed the terminal bud by early November compared to only 11.40% in *P. yunnanensis* and 51.26% in *P. densata* west. At this time of year, the first frosts typically occur in the Pingquan region. Similarly in the Linzhi and Kunming sites, local populations continued to grow in the fall, whereas the northern populations had stopped early.

## Discussion

### CROSSABILITY BARRIER BETWEEN THE THREE PINE SPECIES

Controlled crossing experiments are a direct approach to investigation of reproductive isolation and estimate the strength of genetic barriers between lineages. Our crossing experiments provided comprehensive empirical data on cross-compatibility among the three pine species. First, *P. tabuliformis* and *P. yunnanensis* can cross successfully and yield viable seeds. The mean crossability between the two species was estimated as 0.23 (0.31 based on the method of Critchfield 1967). This crossability is lower than those reported for between three Eurasian pines *P. nigra*, *P. thunbergii*, and *P. tabuliformis* (Kormutak et al. 1992), but typical for most crossable pine species (Critchfield 1975). There is a probability that our crossabilities were underestimated due to the different pollination environments between OP and cross-pollination. The use of stored pollen in interspecific crosses, even though pollen viability was controlled prior to pollination, might present a negative factor in seed production. However, because pollen treatment was the same for all crosses in our experiments, the patterns of crossability between species should not have been affected. Although the crossability between *P. tabuliformis* and *P. yunnanensis* is not very high, it should be sufficient to ensure the production of spontaneous hybrids when the two species were in close parapatry. The contemporary distribution of *P. tabuliformis* and *P. yunnanensis* are allopatric. However, the DNA marker based analyses suggest that *P. yunnanensis* previously occurred in the extant northeastern region of *P. densata*'s range and that it hybridized with *P. tabuliformis* in places where they overlapped, as both of these species appear to have acted as maternal parents (Wang et al. 2011). Significant increases in the altitude of the eastern Tibetan Plateau are thought to have occurred approximately 8 million years ago (Harrison et al. 1992; An et al. 2001), and drastic geographic and climatic changes during the Plio-Pleistocene could have altered the regional flora and separated overlapping or parapatric species. Although hybridization could have occurred in the previously overlapping zone between *P. yunnanensis* and *P. tabuliformis*, the uplift of the eastern Tibetan Plateau and associated climate changes could have gradually pushed the northern edge of the *P. yunnanensis* range southward to its present-day distribution, and populations in the ancestral hybrid zone became fragmented and isolated (Wang et al. 2011; Gao et al. 2012). Our crossing results lend support to the possibility of reciprocal crosses between *P. tabuliformis* and *P. yunnanensis*.

Second, the crossability varied considerably among species combinations and both the maternal and paternal effects were significant in determining the seed efficiency of the interspecific combinations. The mean crossability between *P. densata* and *P. tabuliformis* was 0.39, and between *P. densata* and *P. yunnanensis* was 0.51, both of which were higher than that between *P. tabuliformis* and *P. yunnanensis* at 0.23. This variation in crossability seemed to corroborate with the levels of genetic differentiation between the species (populations). The *P. densata* population selected for this crossing experiment is located in the western range of the species. Our previous studies established that from the ancestral hybrid zone between the two parental species in the northeastern periphery of *P. densata* range, and the species colonized the plateau by stepwise westward migration (Wang et al. 2011; Gao et al. 2012). The direction and intensity of introgression from parental species varied among geographic regions. The western part of the species range was established by limited founders from the central region and has been isolated from *P. yunnanensis* for at least 0.3 million years and from *P. tabuliformis* for at least 6.6 million years (Gao et al. 2012). This resulted in smaller genetic divergence between the western populations of *P. densata* and *P. yunnanensis* (*F_ST_* = 0.14 and 0.10 for cpDNA and nuclear DNA, respectively) compared to *P. tabuliformis* (*F_ST_* = 0.30 and 0.21 for cpDNA and nuclear DNA, respectively; Table4). The correlation between genetic distance and the degree of crossability is suggestive of accumulation of more genetic barriers between *P. densata* and *P. tabuliformis* in the absence of gene flow over a prolonged period of time. Incompatibility between species could be caused by pollen–ovule interaction, cytonuclear interaction, or a combination of these factors (Tiffin et al. 2001; Turelli and Moyle 2007; Lowry et al. 2008). In interspecific crosses between species of subgenus *Pinus*, incompatibility may occur at almost any stage, from the failure of the pollen to germinate to embryo development (McWilliam 1959; Critchfield 1975). Complete inhibition of pollen tube growth on the surface of the nucellus represents the most extreme form of incompatibility among widely divergent species. In this study, we could not unequivocally establish pre- or postfertilization cues for the observed reproductive failure in each cross. The lengthy reproductive process in pines complicates the identification of the genetic mechanism involved in this process.

**Table 4:** Pairwise population differentiation (*F_ST_*) between *Pinus densata* groups and *P. tabuliformis* and *P. yunnanensis* at nuclear and cpSSR (in parentheses) loci

	*P. densata* east	*P. densata* central	*P. densata* west	*P. tabuliformis*	*P. yunnanensis*
*P. densata* east	–				
*P. densata* central	0.23** (0.33**)	–			
*P. densata* west	0.21** (0.32**^)^	0.03 (0.05**)	–		
*P. tabuliformis*	−0.01 (0.01)	0.21** (0.31**)	0.21** (0.30**)	–	
*P. yunnanensis*	0.23** (0.36**)	0.05** (0.04**)	0.10^*^^*^ (0.14**)	0.23** (0.36**)	–

** *P* < 0.01.

In the genus *Pinus*, crossability has been used as a criterion for defining taxonomic relationships (Duffield 1952; Critchfield 1967; Kriebel 1972). In sunflowers, crossing experiments also show that genomic relationships can act as a sensitive predictor of reproductive compatibility (Rieseberg 2000). However, the evolution of genetic barriers in *Pinus* is slow compared to that in other plant genera, especially in cases of speciation via allopolyploidization, which achieve reproductive isolation in two generations. Complete isolation in *Pinus* is observed only among the subsections (Critchfield 1975), which diverged ∼20 million years ago (Willyard et al. 2007). The weak genetic barrier in conifers is attributed to the conservation in genome structure resulting from the paucity of genome rearrangements and lack of whole-genome duplication (Pavy et al. 2012; Nystedt et al. 2013). All *Pinus* species have highly conserved karyotypes with the same 2*n* = 24 chromosomes (Saylor 1972). For these reasons, natural hybrid zones between closely related pine species are frequently detected (Wagner et al. 1987; Edwards-Burke et al. 1997; Watano et al. 2004; Vasilyeva and Goroshkevich 2013), and introgression through backcrossing can easily occur if no other isolation components are acting in the system. Thus, additional reproductive isolation barriers require further study to better understand the evolution of biodiversity in the genus.

### LOCAL ADAPTATION AS AN IMPORTANT COMPONENT IN REPRODUCTIVE ISOLATION

Ecological niche modeling revealed distinct niche shifts among the three pine species (Mao and Wang 2011). *Pinus yunnanensis* occupies a niche with a mild, moist, and low seasonality climate. In contrast, *P. tabuliformis* has a niche with a more continental, arid climate of strong seasonality. The *P. densata* niche is characterized as having high ground frost frequency and diurnal temperature variability, as well as low cumulative heat. Habitat divergence could promote divergent adaptation. Our transplantation experiments revealed the extent of fitness differentiation in this species complex at the early seedling stages.

First, *P. tabuliformis* and *P. yunnanensis* showed distinct habitat preferences, as they each performed better in their respective climate environments. This is particularly evident at the *P. tabuliformis* site Pingquan, where all populations of *P. yunnanensis* were eliminated after the first winter. Large variations in survival and growth within *P. tabuliformis* were also observed, as the southern populations grew relatively poorly at this northern testing site. In the *P. yunnanensis* habitat Kunming, both species showed comparatively high and constant survival rates over the seasons, likely due to the mild and less seasonal climates of the region. Regarding growth, *P. yunnanensis* was 1.8-fold as tall as *P. tabuliformis* by the third year. This gave *P. yunnanensis* a considerable advantage (1.7-fold) in gross population performance over *P. tabuliformis*.

Second, the three groups of *P. densata* differed markedly in performance at each testing site, and this variation closely reflects the genetic composition of the groups. The eastern populations that showed little differentiation from *P. tabuliformis* at nuclear and cpDNA markers (Table4), due to pollen-mediated introgression, survived relatively well at the *P. tabuliformis* site Pingquan compared to *P. densata* central and western groups. At the *P. densata* site Linzhi, although all populations suffered high mortality, the survival rate of the local group, *P. densata* west, was 1.6- to 2.9-fold that of the other two groups of *P. densata*, 2.1-fold that of *P. tabuliformis*, and 2.6-fold that of *P. yunnanensis* by the third year (Table3). This best-performing group at Linzhi site, however, was eliminated at the Pingquan site after the first winter, and showed the lowest germination and fitness at the Kunming site. The *P. densata* central group with low genetic differentiation from *P. yunnanensis* showed similar adaptation as *P. yunnanensis* in Linzhi. Likewise, *P. densata* east had comparable performance as *P. tabuliformis* at this site. These observations suggest that local adaptation in the western range of *P. densata* is strong, and introgression will have negative fitness effect in this environment. Backcrosses between *P. densata* and its parental species (mainly concerns *P. yunnanensis* due to geographical proximity) will be selected against in this habitat due to decline in fitness.

The observed tendency that hybrid populations that are genotypically more similar to one of the parents generally display equivalent fitness components as compared to the parental form is applicable to many plant hybrid systems (Jenczewski et al. 2003). However, genetic distance based on neutral markers does not always correlate with fitness differentiation, as exemplified in *P. densata* central and western groups. The two groups differed little in genetic composition (cpDNA *F_ST_* = 0.05, nuclear *F_ST_* = 0.03, Table4) and yet the fitness of the western group is more than threefold that of the central group (Table3) at the Linzhi site. This discrepancy illustrates that divergent selection may not be detectable at neutral genetic markers, and a failure to detect the expected signature of ecological selection does not necessarily mean that it is absent. As shown by a simulation, the use of neutral markers that are unlinked to selected loci greatly reduces the chance of detecting such signatures (Thibert-Plante and Hendry 2010). Strong differentiation is only expected for genes that are tightly associated with loci contributing to reduced fitness especially in the early stages of ecological speciation (Feder et al. 2012). Thus, the weak differentiation between the western and central groups at the limited neutral marker loci used in this study would only reflect the short divergence time between the two groups. This is an area where genome scans will become particularly informative.

The significant divergence in population performance among the testing sites is readily explained by the lack of adaptation to cope with the extreme environmental conditions in Linzhi and Pingquan, including frost and heat and water availabilities (Mao and Wang 2011). Common garden experiments for conifer trees show that when southern populations are transferred to the north, they suffer frost damage due to a failure to stop growth at the appropriate time, whereas the transfer from north to south results in growth disadvantages due to early bud set and the inability to use the full growth season (Howe et al. 2003; De La Torre et al. 2014). Bud set timing shows clinal variation with respect to latitudinal cline, and occurs as a genetically determined response to photoperiod, temperatures, and drought (Hurme et al. 1997; Alberto et al. 2013). The three species involved in this study are distributed from 24°N to 42°N, which is sufficiently wide to show pronounced divergent responses at each testing site. At the Pingquan site, *P. yunnanensis* and *P. densata* west and central showed low rates of bud set by late autumn, when frost is expected in the region. This could be a direct cause of the high mortality of these southern populations at this site. Similarly, at the Kunming and Linzhi sites, local populations continue to grow in late fall whereas *P. tabuliformis* stopped early. Thus, the higher survival and faster growth of local populations at each site gives them a specific habitat-bounded fitness advantage during the seedling stage. The responses seen in the gardens may have been more pronounced in natural situations where seedling densities may have been higher producing more competition. In forest ecosystems, an advantage at the early seedling stage is a critical lifetime fitness component as it ensures the competitiveness and dominance of the species or lineage in the community.

## Conclusions

The weak genetic barrier but strong juvenile selection suggests that ecological selection played a significant role in the hybrid speciation of *P. densata*. The three pine species exhibit manifest adaptive divergence based on the reduced population fitness in non-native environments. This suggests that premating isolation through selection against immigrants from other habitat types or postzygotic isolation through selection against backcrosses among the three species is strong. A combination of local adaptation and geographic isolation could have maintained and reinforced the interspecific differentiation of these pine species. Overall, our results and other studies (Hurme et al. 1997; Howe et al. 2003; De La Torre et al. 2014) suggest that the conifer species evolve as a direct consequence of adaptation to local environments. The three *P. densata* groups can be regarded as three genetic linages with distinct genetic affiliation and adaptive properties. Because two of the garden sites, Pingquan and Linzhi, were placed in the northern and western ranges of *P. tabuliformis* and *P. densata*, the strong adaptive responses observed among populations and species are not surprising. To better delimit the effect of selection in the face of gene flow, placing additional gardens in geographical regions where introgression are more likely would be informative. Additionally, efforts to identify genomic elements underlying adaptation to the extreme environments on the Tibetan Plateau in conjunction with experiments on hybrid species performance are the next steps in evaluating the genetic basis of ecological divergence operating in this system. Such analyses will continue to provide important data on both the nature of postmating reproductive barriers as well as the genetic basis of adaptation in the hybrid lineages.

## DATA ARCHIVING

The doi for our data is 10.5061/dryad.bt7vr and 10.5061/dryad.566n8.

## Supporting Information

Disclaimer: Supplementary materials have been peer-reviewed but not copyedited.

Table S1 Geographic origins of the 24 populations of *Pinus densata*, *P. tabuliformis*, and *P. yunnanensis* included in the transplantation experiments and the sample size of each population used in nuclear and cpDNA analyses. **Table S2**Interspecific crossabilities (means ± SE) among *Pinus densata*, *P. tabuliformis*, and *P. yunnanensis*. **Table S3**Average survival and height of *Pinus tabuliformis*, *P. yunnanensis*, and the three *P. densata* groups, east, central, and west, at the three plantation sites, Pingquan, Linzhi, and Kunming, over three-year period. **Table S4**Survival and height of the 24 populations at the three plantation sites, Pingquan, Linzhi, and Kunming, over three-year period. **Figure S1**Error-bar chart (mean ± SE) for the five seed and cone morphometric traits, cone length, number of fertile scales per cone, total number of scales per cone, seed length, and seed weight in interspecific crosses and open pollination. **Figure S2**Bar chart of the third year survival, height, and relative fitness of the 24 populations at the three plantation sites.

## References

[b1] Abbott RJ (1992). Plant invasions, interspecific hybridization and the evolution of new plant taxa. Trends Ecol. Evol.

[b2] Alberto FJ, Aitken SN, Alía R, González-Martínez SC, Hänninen H, Kremer A, Lefèvre F, Lenormand T, Yeaman S, Whetten R (2013). Potential for evolutionary responses to climate change evidence from tree populations. Glob. Change Biol.

[b3] An Z, Kutzbach JE, Prell WL, Porter SC (2001). Evolution of Asian monsoons and phased uplift of the Himalayan Tibetan Plateau since late Miocene times. Nature.

[b4] Anderson E, Stebbins GL (1954). Hybridization as an evolutionary stimulus. Evolution.

[b5] Arnold ML (1997). Natural hybridization and evolution.

[b6] Buerkle CA, Morris RJ, Asmussen MA, Rieseberg LH (2000). The likelihood of homoploid hybrid speciation. Heredity.

[b7] Coyne JA, Orr HA (2004). Speciation.

[b8] Critchfield WB (1967). Crossability and relationships of the closed cone pines. Silvae Genet.

[b9] Critchfield WB, Fowler DP, Yeatman CW (1975). Interspecific hybridization in *Pinus*: a summary review. Symposium on interspecific and interprovenance hybridization in forest trees.

[b10] De La Torre AR, Wang T, Jaquish B, Aitken SN (2014). Adaptation and exogenous selection in a *Picea glauca* × *Picea engelmannii* hybrid zone: implications for forest management under climate change. New Phytol.

[b11] De Mendiburu F (2009). Agricolae: statistical procedures for agricultural research. R package version 1.1–2.. http://CRAN.R-project.org/package=agricolae.

[b12] Duffield JW (1952). Relationships and species hybridization in the genus *Pinus*. Silvae Genet.

[b13] Edwards-Burke MA, Hamrick JL, Price RA (1997). Frequency and direction of hybridization in sympatric populations of *Pinus taeda* and *P. echinata* (Pinaceae). Am. J. Bot.

[b14] Excoffier L, Laval G, Schneider S (2005). Arlequin (version 3.0): an integrated software package for population genetics data analysis. Evol. Bioinform.

[b15] Feder JL, Egan SP, Nosil P (2012). The genomics of speciation-with-gene-flow. Trends Genet.

[b16] Fernando DD, Long SM, Sniezko RA (2005). Sexual reproduction and crossing barriers in white pines: the case between *Pinus lambertiana* (sugar pine) and *P. monticola* (western white pine). Tree Genet. Genomes.

[b17] Gao J, Wang B, Mao JF, Ingvarsson P, Zeng QY, Wang XR (2012). Demography and speciation history of the homoploid hybrid pine *Pinus densata* on the Tibetan Plateau. Mol. Ecol.

[b18] Grant V (1981). Plant speciation.

[b19] Harrison TM, Copeland P, Kidd WSF, Yin A (1992). Raising Tibet. Science.

[b20] Howe GT, Aitken SN, Neale DB, Jermstad KD, Wheeler NC, Chen THH (2003). From genotype to phenotype: unraveling the complexities of cold adaptation in forest trees. Can. J. Bot.

[b21] Hurme P, Repo T, Savolainen O, Pääkkönen T (1997). Climatic adaptation of bud set and frost hardiness in Scots pine (*Pinus sylvestris*. Can. J. For. Res.

[b22] Jenczewski E, Ronfort J, Chèvre AM (2003). Crop-to-wild gene flow, introgression and possible fitness effects of transgenes. Environ. Biosafety Res.

[b23] Kormutak A, Vookova B, Gajdošova A, Salaj J (1992). Hybridological relationships between *Pinus nigra* Arn, *Pinus thunbergii* Parl and *Pinus tabulaeformis* Carrière. Silvae Genet.

[b24] Kriebel HB (1972). Embryo development and hybridity barriers in the white pines (Section *Strobus*. Silvae Genet.

[b25] Lowry DB, Modliszewski JL, Wright KM, Wu CA, Willis JH (2008). The strength and genetic basis of reproductive isolating barriers in flowering plants. Philos. Trans. R. Soc. Lond. B Biol. Sci.

[b26] Ma XF, Szmidt AE, Wang XR (2006). Genetic structure and evolutionary history of a diploid hybrid pine *Pinus densata* inferred from the nucleotide variation at seven gene loci. Mol. Biol. Evol.

[b28] Mao JF, Wang XR (2011). Distinct niche divergence characterizes the homoploid hybrid speciation of *Pinus densata* on the Tibetan Plateau. Am. Nat.

[b27] Mao JF, Li Y, Wang XR (2009). Empirical assessment of the reproductive fitness components of the hybrid pine *Pinus densata* on the Tibetan Plateau. Evol. Ecol.

[b29] McWilliam JR (1959). Interspecific incompatibility in *Pinus*. Am. J. Bot.

[b30] Mirov NT (1967). The genus *Pinus*.

[b31] Nystedt B, Street NR, Wetterbom A, Zuccolo A, Lin YC, Scofield DG, Vezzi F, Delhomme N, Giacomello S, Alexeyenko A (2013). The Norway spruce genome sequence and conifer genome evolution. Nature.

[b32] Oksanen J, Blanchet FG, Kindt R, Legendre P, Minchin PR, O'Hara RB, Simpson GL, Solymos P, Stevens MHH, Wagner H (2013). Vegan: community ecology package. R package version 2.0–8.. http://CRAN.R-project.org/package=vegan.

[b33] Park YS, Fowler DP (1984). Inbreeding in black spruce (*Picea mariana* (Mill.) B.S.P.): self-fertility, genetic load, and performance. Can. J. For. Res.

[b34] Pavy N, Pelgas B, Laroche J, Rigault P, Isabel N, Bousquet J (2012). A spruce gene map infers ancient plant genome reshuffling and subsequent slow evolution in the gymnosperm lineage leading to extant conifers. BMC Biol.

[b35] Rieseberg LH (1997). Hybrid origins of plant species. Annu. Rev. Ecol. Syst.

[b36] Rieseberg LH (2000). Crossing relationships among ancient and experimental sunflower hybrid lineages. Evolution.

[b38] Rieseberg LH, Willis JH (2007). Plant speciation. Science.

[b37] Rieseberg LH, Raymond O, Rosenthal DM, Lai Z, Livingstone K, Nakazato T, Durphy JL, Schwarzbach AE, Donovan LA, Lexer C (2003). Major ecological transitions in wild sunflowers facilitated by hybridization. Science.

[b39] Saylor LC (1972). Karyotype analysis of the genus *Pinus*-subgenus *Pinus*. Silvae Genet.

[b40] Schluter D (2001). Ecology and the origin of species. Trends Ecol. Evol.

[b41] Thibert-Plante X, Hendry AP (2010). When can ecological speciation be detected with neutral loci?. Mol. Ecol.

[b42] Tiffin P, Olson MS, Moyle LC (2001). Asymmetrical crossing barriers in angiosperms. Proc. R. Soc. Lond. B Biol. Sci.

[b43] Turelli M, Moyle LC (2007). Asymmetric postmating isolation: Darwin's corollary to Haldane's rule. Genetics.

[b44] Vasilyeva GV, Goroshkevich SN (2013). Crossability of *Pinus sibirica* and *P. pumila* with their hybrids. Silvae Genet.

[b45] Wagner DB, Furnier GR, Saghai-Maroof MA, Williams SM, Dancik BP, Allard RW (1987). Chloroplast DNA polymorphisms in lodgepole and jack pines and their hybrids. Proc. Natl. Acad. Sci. USA.

[b47] Wang XR, Szmidt AE (1994). Hybridization and chloroplast DNA variation in a *Pinus* species complex from Asia. Evolution.

[b48] Wang XR, Szmidt AE, Savolainen O (2001). Genetic composition and diploid hybrid speciation of a high mountain pine, *Pinus densata*, native to the Tibetan plateau. Genetics.

[b46] Wang B, Mao JF, Gao J, Zhao W, Wang XR (2011). Colonization of the Tibetan Plateau by the homoploid hybrid pine *Pinus densata*. Mol. Ecol.

[b49] Watano Y, Kanai A, Tani N (2004). Genetic structure of hybrid zones between *Pinus pumila* and *P. parviflora* var. *Pentaphylla* (Pinaceae) revealed by molecular hybrid index analysis. Am. J. Bot.

[b50] Willyard A, Syring J, Gernandt DS, Liston A, Cronn R (2007). Fossil calibration of molecular divergence infers a moderate mutation rate and recent radiations for *Pinus*. Mol. Biol. Evol.

[b51] Wolf DE, Takebayashi N, Rieseberg LH (2001). Predicting the risk of extinction through hybridization. Conserv. Biol.

