# Supplementary material for: WEAK CROSSABILITY BARRIER BUT STRONG JUVENILE SELECTION SUPPORTS ECOLOGICAL SPECIATION OF THE HYBRID PINE PINUS DENSATA ON THE TIBETAN PLATEAU
Source: Evolution. 2014 Sep 22;68(11):3120–33. doi: 10.1111/evo.12496 (PMC4278550; doi:10.1111/evo.12496)
Supplement: Table S1 — Geographic origins of the 24 populations of Pinus densata, P. tabuliformis, and P. yunnanensis included in the transplantation experiments and the sample size of each population used in nuclear and cpDNA analyses. Table S2Interspecific crossabilities (means ± SE) among Pinus densata, P. tabuliformis, and P. yunnanensis. Table S3Average survival and height of Pinus tabuliformis, P. yunnanensis, and the three P. densata groups, east, central, and west, at the three plantation sites, Pingquan, Linzhi, and Kunming, over three-year period. Table S4Survival and height of the 24 populations at the three plantation sites, Pingquan, Linzhi, and Kunming, over three-year period. Figure S1Error-bar chart (mean ± SE) for the five seed and cone morphometric traits, cone length, number of fertile scales per cone, total number of scales per cone, seed length, and seed weight in interspecific crosses and open pollination. Figure S2Bar chart of the third year survival, height, and relative fitness of the 24 populations at the three plantation sites. [file evo0068-3120-SD1.pdf]

Table S1. Geographic origins of the 24 populations of *Pinus densata*, *Pinus tabuliformis* and *Pinus yunnanensis* included in the transplantation experiments and the sample size of each population used in nuclear and cpDNA analyses.

Species	Population		Longitude (E)	Latitude (N)	Altitude (m)	cpDNA*	Nuclear DNA**
P. densata east	1	Maerkang	102°12'	31°55'	2709	16	10
	2	Lixian	102°48'	31°40'	2765	32	8
	3	Baoxing	102°43'	30°45'	2330	16	12
P. densata central	4	Kangding	101°55'	30°11'	2944	16	10
	5	Zayu	97°13'	29°08'	3264	48	19
	6	Palung Zangbo	95°42'	29°52'	2804	48	18
P. densata west	7	Niyang valley	94°10'	29°45'	3203	96	19
	8	Yarlung Zangbo	94°14'	29°14'	2960	48	19
P. tabuliformis	9	Jiuzhaigou	103°48'	33°18'	2393	na	na
	10	Huzhu	102°27'	36°57'	2300	8	10
	11	Ningshan	108°23'	33°28'	1423	16	9
	12	Lushi	110°49'	33°44'	1713	na	na
	13	Lingkongshan	112°02'	36°36'	1664	8	9
	14	Fangshan	111°33'	37°56'	1941	na	na
	15	Temote	111°13'	40°48'	1219	na	na
	16	Songshan	115°49'	40°31'	855	8	na
	17	Ningcheng	118°58'	42°16'	1300	8	10
P. yunnanensis	18	Zhongdian	99°32'	28°09'	3048	32	na
	19	Lijiang	100°13'	26°53'	2493	16	10
	20	Gongshan	98°49'	25°58'	1616	16	10
	21	Baoshan	99°08'	24°28'	1897	16	na
	22	Kunming	102°37'	24°58'	2242	16	10
	23	Yiliang	103°10'	24°43'	1846	16	na
	24	Yuxi	102°09'	24°15'	1849	16	10

na, DNA data not available.

* data were retrieved from Wang et al. (2011) and Gao et al. (2012).

** data were retrieved from Gao et al. (2012).

Table S2. Interspecific crossabilities (means ± SE) among *Pinus densata*, *Pinus tabuliformis* and *Pinus yunnanensis.*

Maternal species	Paternal species	Crossability[1]	Significance	Crossability[2]	Significance
P. tabuliformis	*P. yunnanensis*	0.21 ±0.02	n.s.	0.28 ±0.02	n.s.
P. yunnanensis	*P. tabuliformis*	0.24 ±0.04		0.35 ±0.04	
	Mean	0.23		0.31	
P. densata	*P. tabuliformis*	0.32 ±0.07	**	0.40 ±0.05	***
P. tabuliformis	*P. densata*	0.46 ±0.02		0.69 ±0.02	
	Mean	0.39		0.55	
P. densata	*P. yunnanensis*	0.63 ±0.09	**	0.86 ±0.08	*
P. yunnanensis	*P. densata*	0.40 ±0.04		0.65 ±0.04	
	Mean	0.51		0.75	

n.s., not significant; * $P < 0.05$; ** $P < 0.01$; *** $P < 0.001$.

[1] Calculated following the definition of this study.

[2] Calculated following the definition of Critchfield (1967).

Table S3. Average survival and height of *P. tabuliformis* , *P. yunnanensis* and the three *P. densata* groups, east, central and west, at the three plantation sites, Pingquan, Linzhi and Kunming, over three year period.

			Survival (%)		Height (cm)	
Site	Time	Species	Mean	SE	Mean	SE
Pingquan	1st Spring	*P. tabuliformis*	[a]62.45	2.87	/	/
		P. densata east	[a]59.58	8.78	/	/
		P. densata central	[b]34.44	1.55	/	/
		P. densata west	[b]38.54	0.21	/	/
		P. yunnanensis	[b]42.32	3.61	/	/
	1st Fall	*P. tabuliformis*	[a]65.69	3.15	[a]8.28	0.44
		P. densata east	[a]68.05	2.89	[a]8.34	0.67
		P. densata central	[b]35.69	3.06	[a]7.61	0.75
		P. densata west	[b]33.75	5.00	[a]7.14	0.14
		P. yunnanensis	[b]21.43	6.44	[a]6.82	0.95
	2nd Spring	*P. tabuliformis*	[a]61.11	2.35	[a]14.03	1.06
		P. densata east	[a]46.94	18.93	[a]12.52	2.34
		P. densata central	[b]0.83	0.83	[a]8.79	na
		P. densata west	[b]0	0	na	na
		P. yunnanensis	[b]0	0	na	na
	2nd Fall	*P. tabuliformis*	[a]50.28	2.36	[a]17.53	1.35
		P. densata east	[a]36.81	16.29	[a]15.59	2.87
		P. densata central	[b]0	0	na	na
		P. densata west	[b]0	0	na	na
		P. yunnanensis	[b]0	0	na	na
	3rd Spring	*P. tabuliformis*	[a]44.03	2.96	[a]21.16	1.65
		P. densata east	[a]31.11	14.38	[a]18.40	4.23
		P. densata central	[b]0	0	na	na
		P. densata west	[b]0	0	na	na
		P. yunnanensis	[b]0	0	na	na
Linzhi	1st Spring	*P. tabuliformis*	[a]82.06	2.66	/	/
		P. densata east	[ab]74.50	7.51	/	/
		P. densata central	[b]60.83	6.41	/	/
		P. densata west	[ab]70.25	0.75	/	/
		P. yunnanensis	[b]62.86	4.70	/	/
	1st Fall	*P. tabuliformis*	[a]40.78	4.06	[a]2.70	0.14
		P. densata east	[a]38.83	9.23	[a]2.66	0.11
		P. densata central	[b]21.00	3.51	[a]2.77	0.52
		P. densata west	[a]38.00	3.50	[a]3.26	0.25
		P. yunnanensis	[ab]25.14	2.32	[a]3.00	0.34
	2nd Spring	*P. tabuliformis*	[b]11.11	1.31	[ab]6.12	0.57
		P. densata east	[b]14.33	6.34	[ab]6.15	0.6
		P. densata central	[b]7.67	3.49	[ab]6.39	0.87
		P. densata west	[a]27.75	3.25	[a]7.77	1.55
		P. yunnanensis	[b]9.57	2.23	[b]5.01	0.4
	2nd Fall	*P. tabuliformis*	[b]10.28	1.09	[b]8.49	0.68
		P. densata east	[b]13.17	5.67	[b]8.69	0.9
		P. densata central	[b]7.00	3.55	[ab]10.85	1.33
		P. densata west	[a]24.50	2.50	[a]12.23	1.26
		P. yunnanensis	[b]9.36	2.20	[ab]9.51	0.68

Table S3 continued

Site	Time	Species	Survival (%)		Height (cm)	
			Mean	SE	Mean	SE
Linzhi	3rd Spring	*P. tabuliformis*	^{b}9.56	1.04	^{ab}12.88	1.23
		P. densata east	^{ab}12.17	4.92	^{b}11.04	1.08
		P. densata central	^{b}6.83	3.38	^{ab}12.51	1.09
		P. densata west	^{a}19.75	4.25	^{a}17.41	1.75
		P. yunnanensis	^{b}7.57	1.94	^{ab}12.89	1.54
Kunming	1st Spring	*P. tabuliformis*	^{a}85.56	1.97	/	/
		P. densata east	^{a}79.67	6.67	/	/
		P. densata central	^{a}73.11	10.17	/	/
		P. densata west	^{b}56.83	7.17	/	/
		P. yunnanensis	^{a}83.92	1.08	/	/
	1st Fall	*P. tabuliformis*	^{a}83.85	1.85	^{a}4.37	0.09
		P. densata east	^{a}77.67	6.83	^{a}4.76	0.23
		P. densata central	^{a}71.78	10.17	^{a}4.47	0.48
		P. densata west	^{b}55.50	6.83	^{a}4.39	0.16
		P. yunnanensis	^{a}81.53	1.33	^{a}5.27	0.61
	2nd Spring	*P. tabuliformis*	^{a}83.44	1.79	^{a}6.94	0.38
		P. densata east	^{a}77.44	7.06	^{a}9.06	0.33
		P. densata central	^{a}71.67	10.14	^{a}9.18	1.29
		P. densata west	^{b}55.00	6.67	^{a}9.32	0.57
		P. yunnanensis	^{a}81.06	1.41	^{a}10.61	1.48
	2nd Fall	*P. tabuliformis*	^{a}82.96	1.73	^{a}8.97	0.55
		P. densata east	^{a}76.67	7.34	^{a}11.33	0.63
		P. densata central	^{a}71.44	10.17	^{a}12.46	2.52
		P. densata west	^{b}54.33	7.33	^{a}12.66	0.54
		P. yunnanensis	^{a}80.72	1.28	^{a}16.00	2.84
	3rd Spring	*P. tabuliformis*	^{a}83.84	1.98	^{b}15.71	0.93
		P. densata east	^{a}76.25	7.34	^{ab}20.01	0.63
		P. densata central	^{a}70.83	10.48	^{ab}20.71	3.47
		P. densata west	^{b}54.17	7.50	^{ab}21.13	0.98
		P. yunnanensis	^{a}78.23	1.23	^{a}27.80	3.28

SE, standard error; /, not measured; na, not available due to mortality.
Means with different superscript letters (a, b) are significantly different ($P = 0.05$) in LSD multiple-range test.

Table S4. Survival and height of the 24 populations at the three plantation sites, Pingquan, Linzhi and Kunming, over three year period.

Site	Traits	Species (Group)	Population		Survival (%)	Height (cm)
Pingquan	1st Spring	*P. densata* east	1	Maerkang	64.59	/
			2	Lixian	71.67	/
			3	Baoxing	42.50	/
		P. densata central	4	Kangding	33.33	/
			5	Zayu	32.50	/
			6	Palung Zangbo	37.50	/
		P. densata west	7	Niyang valley	38.34	/
			8	Yarlung Zangbo	38.75	/
		P. tabuliformis	9	Jiuzhaigou	60.42	/
			10	Huzhu	59.17	/
			11	Ningshan	57.09	/
			12	Lushi	53.33	/
			13	Lingkongshan	70.42	/
			14	Fangshan	49.58	/
			15	Temote	69.17	/
			16	Songshan	75.42	/
			17	Ningcheng	67.50	/
		P. yunnanensis	18	Zhongdian	27.09	/
			19	Lijiang	56.67	/
			20	Gongshan	43.75	/
			21	Baoshan	44.59	/
			22	Kunming	46.67	/
			23	Yiliang	33.33	/
			24	Yuxi	44.17	/
	1st Fall	*P. densata* east	1	Maerkang	72.92	8.62
			2	Lixian	68.33	9.32
			3	Baoxing	62.92	7.06
		P. densata central	4	Kangding	39.17	6.12
			5	Zayu	29.59	8.54
			6	Palung Zangbo	38.33	8.18
		P. densata west	7	Niyang valley	28.75	7.28
			8	Yarlung Zangbo	38.75	7.00
		P. tabuliformis	9	Jiuzhaigou	62.50	9.38
			10	Huzhu	44.58	7.35
			11	Ningshan	66.67	9.22
			12	Lushi	63.75	9.41
			13	Lingkongshan	78.34	7.20
			14	Fangshan	72.50	6.35
			15	Temote	72.08	9.11
			16	Songshan	63.34	9.68
			17	Ningcheng	67.50	6.83
		P. yunnanensis	18	Zhongdian	26.67	7.53
			19	Lijiang	52.09	4.42
			20	Gongshan	26.67	7.13
			21	Baoshan	2.92	11.82
			22	Kunming	23.33	5.84
			23	Yiliang	16.25	4.54
			24	Yuxi	2.08	6.48

Table S4 continued

Site	Traits	Species (Group)	Population		Survival (%)	Height (cm)
Pingquan	2nd Spring	*P. densata* east	1	Maerkang	67.92	14.66
			2	Lixian	63.75	15.07
			3	Baoxing	9.17	7.84
		P. densata central	4	Kangding	0	na
			5	Zayu	2.50	8.79
			6	Palung Zangbo	0	na
		P. densata west	7	Niyang valley	0	na
			8	Yarlung Zangbo	0	na
		P. tabuliformis	9	Jiuzhaigou	54.59	9.74
			10	Huzhu	56.67	13.78
			11	Ningshan	51.25	9.22
			12	Lushi	58.33	12.45
			13	Lingkongshan	71.67	14.37
			14	Fangshan	70.83	14.43
			15	Temote	65.83	18.51
			16	Songshan	59.59	17.46
			17	Ningcheng	61.25	16.35
		P. yunnanensis	18	Zhongdian	0	na
			19	Lijiang	0	na
			20	Gongshan	0	na
			21	Baoshan	0	na
			22	Kunming	0	na
			23	Yiliang	0	na
			24	Yuxi	0	na
	2nd Fall	*P. densata* east	1	Maerkang	57.08	18.42
			2	Lixian	48.75	18.50
			3	Baoxing	4.58	9.86
		P. densata central	4	Kangding	0	na
			5	Zayu	0	na
			6	Palung Zangbo	0	na
		P. densata west	7	Niyang valley	0	na
			8	Yarlung Zangbo	0	na
		P. tabuliformis	9	Jiuzhaigou	43.75	11.78
			10	Huzhu	52.08	18.17
			11	Ningshan	37.08	11.13
			12	Lushi	50	16.39
			13	Lingkongshan	50	18.63
			14	Fangshan	62.92	17.72
			15	Temote	51.25	23.17
			16	Songshan	54.59	22.08
			17	Ningcheng	50.84	18.71
		P. yunnanensis	18	Zhongdian	0	na
			19	Lijiang	0	na
			20	Gongshan	0	na
			21	Baoshan	0	na
			22	Kunming	0	na
			23	Yiliang	0	na
			24	Yuxi	0	na

Table S4 continued

Site	Traits	Species (Group)	Population		Survival (%)	Height (cm)
Pingquan	3rd Spring	*P. densata* east	1	Maerkang	47.92	22.73
			2	Lixian	42.92	22.52
			3	Baoxing	2.50	9.94
		P. densata central	4	Kangding	0	na
			5	Zayu	0	na
			6	Palung Zangbo	0	na
		P. densata west	7	Niyang valley	0	na
			8	Yarlung Zangbo	0	na
		P. tabuliformis	9	Jiuzhaigou	33.75	13.96
			10	Huzhu	47.92	23.88
			11	Ningshan	26.25	12.30
			12	Lushi	40.84	21.22
			13	Lingkongshan	47.50	21.77
			14	Fangshan	54.17	21.87
			15	Temote	50.42	27.32
			16	Songshan	48.33	25.24
			17	Ningcheng	47.09	22.92
		P. yunnanensis	18	Zhongdian	0	na
			19	Lijiang	0	na
			20	Gongshan	0	na
			21	Baoshan	0	na
			22	Kunming	0	na
			23	Yiliang	0	na
			24	Yuxi	0	na
Linzhi	1st Spring	*P. densata* east	1	Maerkang	81.50	/
			2	Lixian	82.50	/
			3	Baoxing	59.50	/
		P. densata central	4	Kangding	49.00	/
			5	Zayu	62.50	/
			6	Palung Zangbo	71.00	/
		P. densata west	7	Niyang valley	69.50	/
			8	Yarlung Zangbo	71.00	/
		P. tabuliformis	9	Jiuzhaigou	80.50	/
			10	Huzhu	95.50	/
			11	Ningshan	79.50	/
			12	Lushi	73.50	/
			13	Lingkongshan	90.50	/
			14	Fangshan	72.00	/
			15	Temote	88.00	/
			16	Songshan	76.00	/
			17	Ningcheng	83.00	/
		P. yunnanensis	18	Zhongdian	60.50	/
			19	Lijiang	77.50	/
			20	Gongshan	39.00	/
			21	Baoshan	70.50	/
			22	Kunming	65.50	/
			23	Yiliang	57.50	/
			24	Yuxi	69.50	/

Table S4 continued

Site	Traits	Species (Group)	Population		Survival (%)	Height (cm)
Linzhi	1st Fall	*P. densata* east	1	Maerkang	53.00	2.78
			2	Lixian	42.00	2.43
			3	Baoxing	21.50	2.77
		P. densata central	4	Kangding	14.00	1.91
			5	Zayu	24.00	2.70
			6	Palung Zangbo	25.00	3.72
		P. densata west	7	Niyang valley	41.50	3.51
			8	Yarlung Zangbo	34.50	3.02
		P. tabuliformis	9	Jiuzhaigou	22.50	3.38
			10	Huzhu	48.50	2.40
			11	Ningshan	40	3.33
			12	Lushi	34.00	2.80
			13	Lingkongshan	59.50	2.32
			14	Fangshan	38.50	2.55
			15	Temote	57.50	2.69
			16	Songshan	33.00	2.72
			17	Ningcheng	33.50	2.14
		P. yunnanensis	18	Zhongdian	15.00	2.67
			19	Lijiang	30	2.01
			20	Gongshan	20	3.75
			21	Baoshan	28.50	4.61
			22	Kunming	28.00	3.01
			23	Yiliang	22.50	2.41
			24	Yuxi	32.00	2.56
	2nd Spring	*P. densata* east	1	Maerkang	27.00	6.50
			2	Lixian	7.50	4.98
			3	Baoxing	8.50	6.97
		P. densata central	4	Kangding	3.00	6.39
			5	Zayu	5.50	4.88
			6	Palung Zangbo	14.50	7.90
		P. densata west	7	Niyang valley	31.00	9.32
			8	Yarlung Zangbo	24.50	6.22
		P. tabuliformis	9	Jiuzhaigou	12.00	9.23
			10	Huzhu	8.00	4.41
			11	Ningshan	17.00	7.90
			12	Lushi	14.50	7.49
			13	Lingkongshan	14.00	4.81
			14	Fangshan	5.00	6.40
			15	Temote	11.50	5.02
			16	Songshan	11.50	4.95
			17	Ningcheng	6.50	4.90
		P. yunnanensis	18	Zhongdian	9.50	6.29
			19	Lijiang	16.50	3.85
			20	Gongshan	7.50	5.41
			21	Baoshan	0	na
			22	Kunming	15.00	5.85
			23	Yiliang	13.50	4.57
			24	Yuxi	5.00	4.11

Table S4 continued

Site	Traits	Species (Group)	Population		Survival (%)	Height (cm)
Linzhi	2nd Fall	*P. densata* east	1	Maerkang	24.50	8.28
			2	Lixian	7.00	7.37
			3	Baoxing	8.00	10.42
		P. densata central	4	Kangding	2.50	9.97
			5	Zayu	4.50	9.11
			6	Palung Zangbo	14.00	13.47
		P. densata west	7	Niyang valley	27.00	13.50
			8	Yarlung Zangbo	22.00	10.97
		P. tabuliformis	9	Jiuzhaigou	11.50	12.15
			10	Huzhu	8.00	6.52
			11	Ningshan	14.00	11.24
			12	Lushi	14.00	8.85
			13	Lingkongshan	12.50	6.81
			14	Fangshan	5.00	9.09
			15	Temote	10.50	7.30
			16	Songshan	11.00	6.93
			17	Ningcheng	6.00	7.49
		P. yunnanensis	18	Zhongdian	9.50	9.55
			19	Lijiang	16.50	8.19
			20	Gongshan	7.00	10.50
			21	Baoshan	0	na
			22	Kunming	14.50	12.21
			23	Yiliang	13.00	8.94
			24	Yuxi	5.00	7.68
	3rd Spring	*P. densata* east	1	Maerkang	22.00	12.95
			2	Lixian	7.00	9.23
			3	Baoxing	7.50	10.95
		P. densata central	4	Kangding	2.50	13.08
			5	Zayu	4.50	10.41
			6	Palung Zangbo	13.50	14.05
		P. densata west	7	Niyang valley	24.00	19.16
			8	Yarlung Zangbo	15.50	15.66
		P. tabuliformis	9	Jiuzhaigou	10	17.14
			10	Huzhu	8.00	9.75
			11	Ningshan	14.00	20.62
			12	Lushi	13.50	13.34
			13	Lingkongshan	10.50	11.80
			14	Fangshan	5.00	11.71
			15	Temote	10.50	10.19
			16	Songshan	9.00	11.67
			17	Ningcheng	5.50	9.73
		P. yunnanensis	18	Zhongdian	9.00	14.65
			19	Lijiang	13.00	8.85
			20	Gongshan	5.50	15.34
			21	Baoshan	0	na
			22	Kunming	13.50	17.81
			23	Yiliang	9.50	12.40
			24	Yuxi	2.50	8.27

Table S4 continued

Site	Traits	Species (Group)	Population		Survival (%)	Height (cm)
Kunming	1st Spring	*P. densata* east	1	Maerkang	86.00	/
			2	Lixian	86.67	/
			3	Baoxing	66.33	/
		P. densata central	4	Kangding	53.67	/
			5	Zayu	88.00	/
			6	Palung Zangbo	77.67	/
		P. densata west	7	Niyang valley	49.67	/
			8	Yarlung Zangbo	64.00	/
		P. tabuliformis	9	Jiuzhaigou	90	/
			10	Huzhu	85.00	/
			11	Ningshan	78.67	/
			12	Lushi	74.00	/
			13	Lingkongshan	90.67	/
			14	Fangshan	87.33	/
			15	Temote	89.00	/
			16	Songshan	84.00	/
			17	Ningcheng	91.33	/
		P. yunnanensis	18	Zhongdian	82.33	/
			19	Lijiang	84.45	/
			20	Gongshan	87.33	/
			21	Baoshan	83.33	/
			22	Kunming	81.34	/
			23	Yiliang	80.67	/
			24	Yuxi	88.00	/
	1st Fall	*P. densata* east	1	Maerkang	84.67	5.18
			2	Lixian	84.33	4.39
			3	Baoxing	64.00	4.70
		P. densata central	4	Kangding	52.33	3.54
			5	Zayu	86.67	4.70
			6	Palung Zangbo	76.33	5.17
		P. densata west	7	Niyang valley	48.67	4.23
			8	Yarlung Zangbo	62.33	4.56
		P. tabuliformis	9	Jiuzhaigou	87.67	4.68
			10	Huzhu	84.00	4.36
			11	Ningshan	77.00	4.30
			12	Lushi	72.67	4.86
			13	Lingkongshan	87.33	4.23
			14	Fangshan	86.00	4.16
			15	Temote	87.00	4.45
			16	Songshan	83.33	4.32
			17	Ningcheng	89.67	4.00
		P. yunnanensis	18	Zhongdian	79.67	4.08
			19	Lijiang	79.72	3.46
			20	Gongshan	85.67	6.62
			21	Baoshan	82.00	8.02
			22	Kunming	78.33	4.99
			23	Yiliang	78.33	4.11
			24	Yuxi	87.00	5.60

Table S4 continued

Site	Traits	Species (Group)	Population		Survival (%)	Height (cm)
Kunming	2nd Spring	*P. densata* east	1	Maerkang	84.67	9.08
			2	Lixian	84.33	8.48
			3	Baoxing	63.33	9.63
		P. densata central	4	Kangding	52.33	7.07
			5	Zayu	86.67	8.94
			6	Palung Zangbo	76.00	11.53
		P. densata west	7	Niyang valley	48.33	8.75
			8	Yarlung Zangbo	61.67	9.89
		P. tabuliformis	9	Jiuzhaigou	86.33	7.14
			10	Huzhu	83.33	6.11
			11	Ningshan	77.00	7.04
			12	Lushi	72.67	9.40
			13	Lingkongshan	86.67	6.68
			14	Fangshan	85.33	6.20
			15	Temote	87.00	7.87
			16	Songshan	83.00	6.41
			17	Ningcheng	89.67	5.63
		P. yunnanensis	18	Zhongdian	78.67	8.71
			19	Lijiang	79.72	6.99
			20	Gongshan	85.33	14.40
			21	Baoshan	82.00	17.39
			22	Kunming	77.67	10.28
			23	Yiliang	77.33	6.90
			24	Yuxi	86.67	9.58
	2nd Fall	*P. densata* east	1	Maerkang	83.67	10.91
			2	Lixian	84.33	10.50
			3	Baoxing	62.00	12.57
		P. densata central	4	Kangding	52.00	8.96
			5	Zayu	86.33	11.08
			6	Palung Zangbo	76.00	17.34
		P. densata west	7	Niyang valley	47.00	12.11
			8	Yarlung Zangbo	61.67	13.20
		P. tabuliformis	9	Jiuzhaigou	84.00	9.16
			10	Huzhu	82.33	7.55
			11	Ningshan	77.00	10.66
			12	Lushi	72.67	12.14
			13	Lingkongshan	86.67	8.58
			14	Fangshan	85.00	7.55
			15	Temote	86.67	9.68
			16	Songshan	83.00	8.36
			17	Ningcheng	89.33	7.07
		P. yunnanensis	18	Zhongdian	78.33	13.29
			19	Lijiang	79.72	8.95
			20	Gongshan	84.67	22.25
			21	Baoshan	81.67	29.92
			22	Kunming	77.67	15.08
			23	Yiliang	77.33	9.99
			24	Yuxi	85.67	12.54

Table S4 continued

Site	Traits	Species (Group)	Population		Survival (%)	Height (cm)
Kunming	3rd Spring	*P. densata* east	1	Maerkang	82.08	21.14
			2	Lixian	85.00	18.96
			3	Baoxing	61.67	19.94
		P. densata central	4	Kangding	51.25	14.37
			5	Zayu	87.08	21.44
			6	Palung Zangbo	74.17	26.32
		P. densata west	7	Niyang valley	46.67	20.15
			8	Yarlung Zangbo	61.67	22.11
		P. tabuliformis	9	Jiuzhaigou	82.92	15.83
			10	Huzhu	86.25	11.13
			11	Ningshan	75.00	17.77
			12	Lushi	72.92	19.87
			13	Lingkongshan	87.50	15.71
			14	Fangshan	86.67	13.50
			15	Temote	90.42	17.60
			16	Songshan	85.83	17.21
			17	Ningcheng	87.09	12.77
		P. yunnanensis	18	Zhongdian	77.08	26.31
			19	Lijiang	76.33	21.36
			20	Gongshan	83.34	28.41
			21	Baoshan	77.92	46.02
			22	Kunming	74.58	29.11
			23	Yiliang	76.25	21.31
			24	Yuxi	82.08	22.09

/, not measured; na, not available due to mortality.

Fig. S1

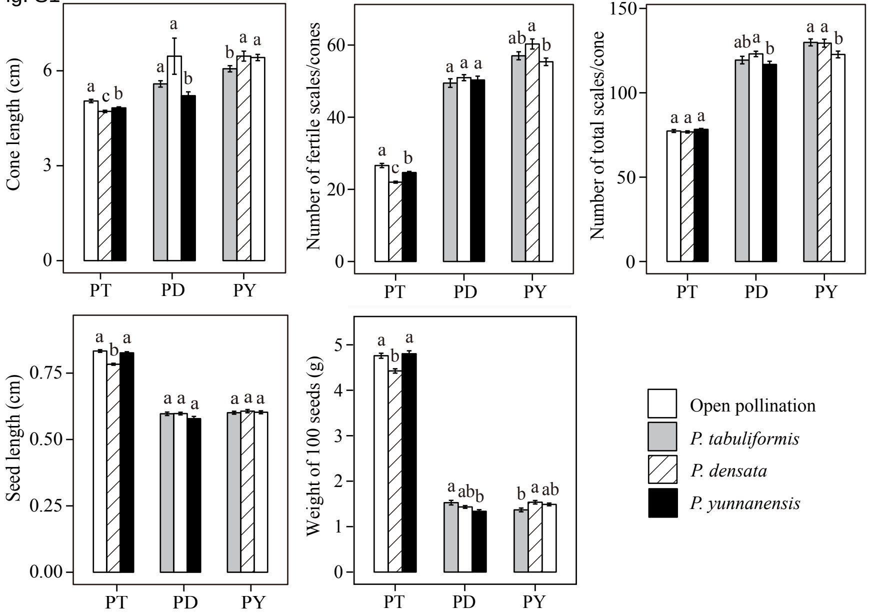
Cone length (cm)
Number of fertile scales/cones
Number of total scales/cone
Seed length (cm)
Weight of 100 seeds (g)
PT
PD
PY
Open pollination
P. tabuliformis
P. densata
P. yunnanensis

Fig. S2

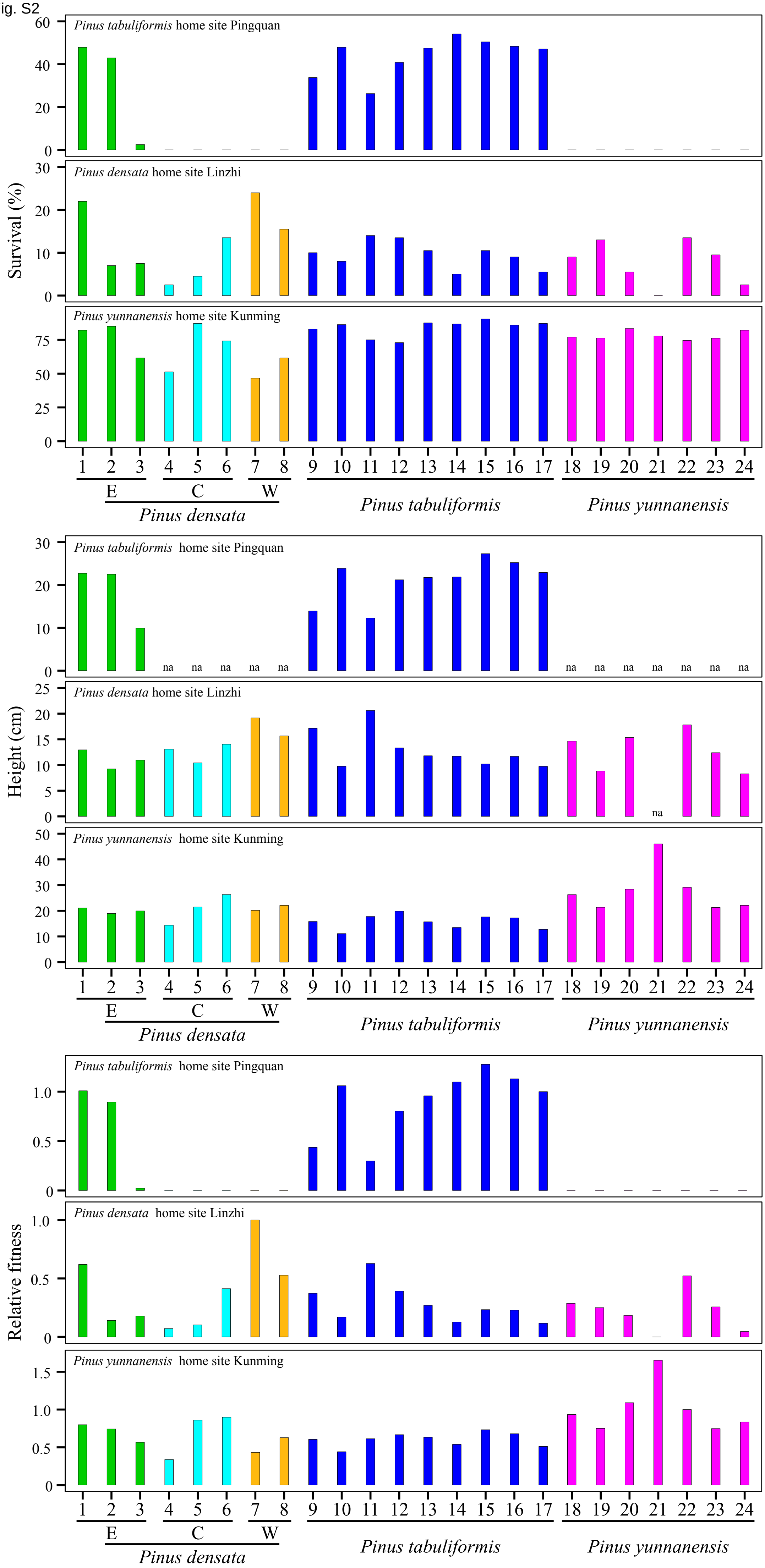

Pinus tabuliformis home site Pingquan
Pinus densata home site Linzhi
Pinus yunnanensis home site Kunming
Survival (%)
Height (cm)
Relative fitness
na
1 2 3 4 5 6 7 8 9 10 11 12 13 14 15 16 17 18 19 20 21 22 23 24
E
C
W
Pinus densata
Pinus tabuliformis
Pinus yunnanensis
